# Hypertensive disorders of pregnancy and stroke: a univariate and multivariate Mendelian randomization study

**DOI:** 10.3389/fendo.2024.1366023

**Published:** 2024-10-21

**Authors:** Kang Qu, Mingxi Li, Peng Yu, Wei Jiang, Ming Dong

**Affiliations:** ^1^ Department of Neurology and Neuroscience Center, The First Hospital of Jilin University, Changchun, China; ^2^ Department of Ophthalmology, The Second Hospital of Jilin University, Changchun, China

**Keywords:** hypertensive disorders of pregnancy, preeclampsia, gestational hypertension, Mendelian randomization, stroke

## Abstract

**Background:**

Hypertensive disorders of pregnancy (HDP) are associated with an increased risk of stroke later in life in multiparous women. However, causality of these associations remains unclear. This study employed 2-sample univariate and multivariate Mendelian randomization (MR) to assess the causal connection between HDP and stroke.

**Methods:**

Genetic variants for HDP and two subtypes were identified from recent large-scale genome-wide association studies and the FinnGen consortium. Stroke summary data were obtained from the MEGASTROKE consortium. The primary analytical approach for univariate MR was the inverse variance weighting method. Sensitivity analyses incorporated methods such as MR-Egger regression, weighted median, and maximum likelihood to ascertain the robustness of the results. Additionally, multivariable MR analyses were conducted to account for potential associative effects of hypertension and type 2 diabetes.

**Results:**

Genetically predicted HDP was associated with a high risk of large artery atherosclerosis (odds ratio [OR]=1.50, 95% confidence interval [CI]: 1.17–1.91, *P*=1.13×10^-3^) and small vessel stroke (OR=1.29, 95% CI: 1.20–1.50, *P*=1.52×10^-3^). HDP may also correlate with ischemic stroke (OR=1.13, 95% CI: 1.04–1.23, *P*=4.99×10^-3^) and stroke (OR=1.11, 95% CI: 1.03–1.20, *P*=8.85×10^-3^). An elevated risk of small vessel stroke (OR=1.20, 95% CI: 1.01–1.43, *P*=3.74×10^-2^) and large artery atherosclerosis (OR=1.22, 95% CI: 1.01–1.47, P=4.07×10^-2^) may be related with genetically predicted susceptibility to gestational hypertension. Genetically predicted susceptibility to preeclampsia or eclampsia may be associated with an increased risk of stroke (OR = 1.10, 95% CI: 1.02–1.19, *P* = 1.16×10^-2^) and ischemic stroke (OR = 1.10, 95% CI: 1.02–1.20, *P* = 1.84×10^-2^). Type 2 diabetes mellitus and hypertension were identified as significant factors contributing to the association between HDP and stroke.

**Conclusions:**

This study provides genetic evidence supporting an association between HDP and increased stroke risk bolstering HDP as a cerebrovascular risk factor.

## Introduction

Hypertensive disorders of pregnancy (HDP) are characterized by hypertension and widespread endothelial dysfunction during pregnancy including gestational hypertension (GH), preeclampsia or eclampsia (PE), chronic hypertension, and chronic hypertension with superimposed preeclampsia (1). An estimated 10%–15% of pregnancies are complicated by HDP, which continues to be one of the leading causes of maternal and fetal morbidity and mortality associated with pregnancy globally (2, 3). HDP can negatively affect multiple organ systems, mother and fetus results, and the mother’s short- and long-term health. There is general agreement that HDP is a risk factor for cardiovascular disease, accounting for approximately 8% of pregnancy-related cardiovascular fatalities (4–6).

Stroke is a leading cause of death and disability worldwide (7). Stroke is characterized by the abrupt onset of neurological deficits, encompassing both ischemic and hemorrhagic strokes (8). Ischemic stroke (IS), according to the etiology of the disease, is classified into five subtypes under the Trial of ORG 10172 in Acute Stroke Treatment criteria (8). The more prevalent subtypes include large artery atherosclerosis (LAA), small vessel stroke (SVS), and cardioembolic stroke (CES). Previous observational data indicate that women with a history of HDP are more likely to have a stroke than women with normotensive pregnancies, and this risk has continued for decades (9, 10). A recent cohort study suggested that women with a history of HDP have a 2.27-fold higher risk of stroke events than healthy pregnant women (11). A recent meta-analysis of 10 million participants indicating that HDP is associated with an increased risk of stroke later in life among multiparous women (12, 13). However, causal inference cannot be concluded from such observational associations owing to the potential residual impact of confounding factors, whether HDP are independent and causal risk factors for stroke are less established. Furthermore, rather than treating stroke as a distinct event, the majority of previous studies have examined stroke as a component of global cardiovascular outcomes (14, 15) or ignored stroke subtypes when analyzing HDP effect on stroke risk,^6^ which could have obscured the actual significance of the association between HDP and stroke.

Mendelian randomization (MR) is a method that uses genetic variants as exposure instrumental variables to assess whether risk factors have causal associations with an outcome of interest (16). Since genetic variants are randomly distributed at conception and are minimally influenced by personality, lifestyle, and environmental confounders, this method may minimize the impact of residual confounding (17).

In this study we conducted a 2-sample MR study to examine the association of genetic predisposition to HDP and its 2 subtypes with stroke and its subtypes. We aimed to provide evidence regarding the causal role of HDP in maternal stroke.

## Methods

### Study design, data availability, and ethics statement

**Figure 1:**
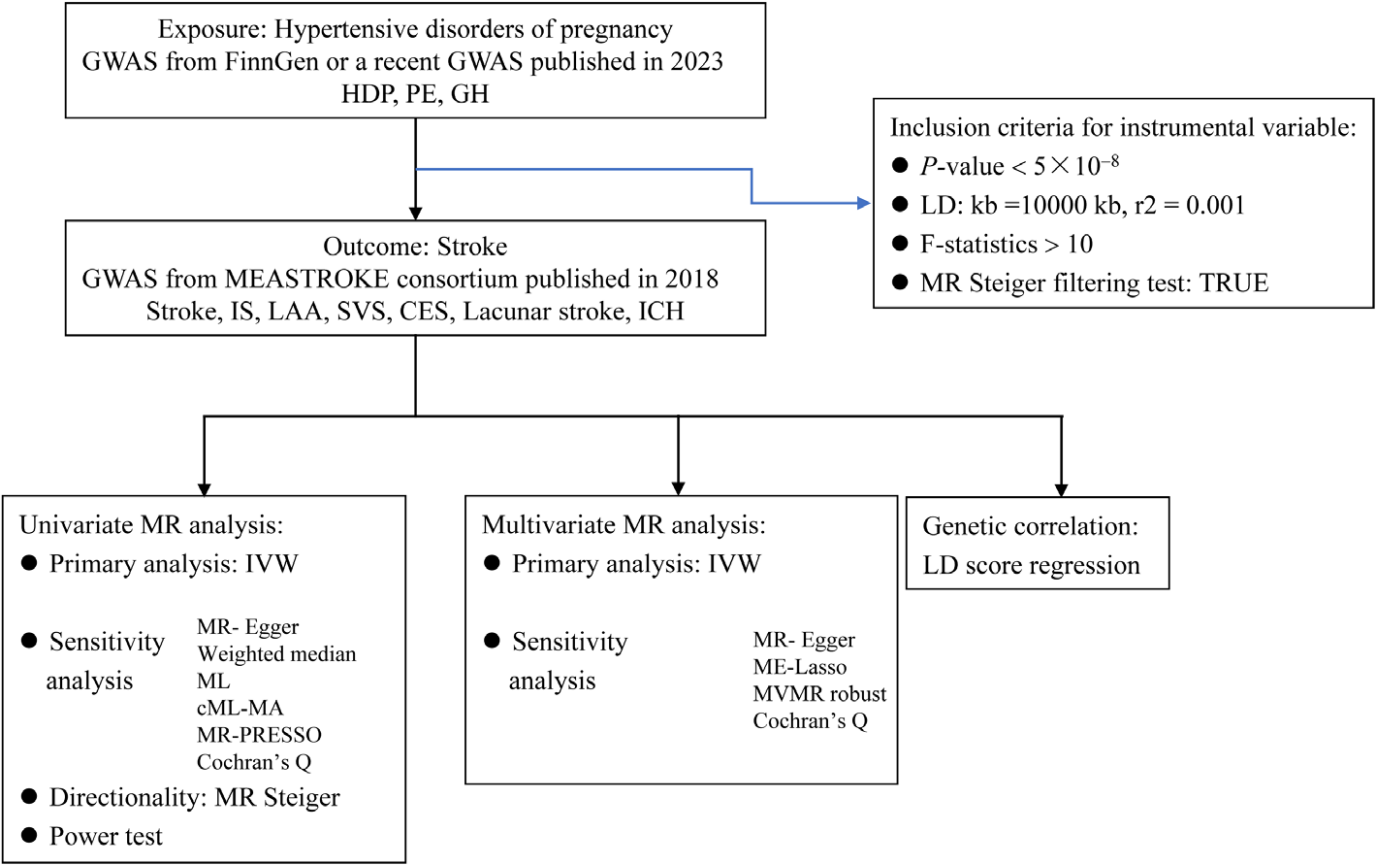
Flow chart of the mendelian randomization study. MR, mendelian randomization; GWAS, genome-wide association study; HDP, hypertensive disorders of pregnancy; PE, preeclampsia; GH, gestational hypertension; IS, ischemic stroke; LAA, large artery atherosclerosis; SVS, small vessel stroke, CES, cardioembolic stroke; ICH, intracranial hemorrhage; LD, linkage disequilibrium; IVW, inverse variance-weighted multiplication; ML, maximum likelihood; CML-MA, constrained ML and model averaging; MR-PRESSO, MR pleiotropy residual sum and outlier; MVMR, multivariate MR.

Our research employed a 2-sample MR design based on the 3 key MR assumptions (18). **Figure 1** illustrates the strategy used in this study. The MR studies were reported in accordance with the suggestions made by Strengthening the Reporting of Observational Studies in Epidemiology Using Mendelian Randomization reporting guidelines (19). No further ethical approval was required because the genome wide association study (GWAS) summary data utilized in this study were already publicly available and the first scientific investigation passed ethical assessment. **Supplementary Table S1** lists the download links for the GWAS summary data used in this study.

### Sources of GWAS data

Pooled GWAS data for HDP (n=296,824) and PE (130,207) were obtained from a recent GWAS meta-analysis involving 4 cohorts (20). In this study, the term ‘PE’ encompasses not only preeclampsia and eclampsia but also chronic hypertension with superimposed preeclampsia. For clarity, all three conditions are referred to as ‘PE’ in the subsequent sections of this paper. The GWAS summary data for GH (n= 202,768) were extracted from the FinnGen Consortium (R9) (21, 22). The MEGASTROKE consortium, which performed a meta-analysis of 29 European-descent GWAS studies, provided a pooled set of GWAS data for stroke, including 40,585 stroke cases and 406,111 controls (8). All stroke cases were confirmed using clinical and imaging criteria. including any stroke. A stroke encompasses all types of strokes, whereas an IS specifically refers to those diagnosed as IS. According to the TOAST classification criteria, IS is categorized into three subtypes: LAA, SVS, and CES. Summary data on lacunar stroke (n= 254,959) were obtained from the most recent GWAS study (23). Lacunar stroke was defined according to the TOAST criteria or brain magnetic resonance imaging. Pooled GWAS data for intracranial hemorrhage (ICH) (n=473,513) were obtained from a study on genetic associations in human disease phenotypes (24). **Supplementary Table S1** provides details of the included GWAS summary data, encompassing disease diagnostic criteria and demographic data. The exposure and outcome datasets had virtually no sample overlap.

### Selection of instrumental variables

To proxy HDP, PE, and GH, single-nucleotide polymorphisms (SNPs) were used as genetic instruments at a genome-wide significance level (*P*<5×10^−8^). We conducted linkage disequilibrium (LD) clumping between SNPs (r^2^ = 0.001, window size=10,000 kb) according to European data from the 1000 Genomes Project. F-statistics were calculated by the formula F=((N−K−1)×R^2^)/(K×(1−R^2^)) (25). R^2^ was the proportion of variation in exposure explained by the genetic instruments, N was the sample size of the exposure GWAS, and K was the number of genetic instruments used. An F-statistic greater than 10 indicated the absence of a weak instrumental variable. SNPs with incompatible alleles, palindromic SNPs with intermediate allele frequencies, and SNPs directly associated with outcome (*P*<5×10^−8^) were excluded. Additionally, the MR Steiger filtering test was used to determine directionality of the exposure effect on outcomes (26). The “TRUE” result predicted the expected effect direction of exposure on the outcome. “FALSE” results represented SNPs that explained a greater proportion of variance in the outcome than in the exposure, and such SNPs were excluded to reduce bias due to reverse-causality instrument variables.

### Mendelian randomization analysis

Two-sample univariate MR analyses were performed separately to estimate the genetically predicted effects of exposure on outcomes. The random effects inverse variance-weighted multiplication (IVW) method, which assumes that all SNPs are valid instruments and allows for pleiotropy while providing the most precise estimates, was used as the primary MR analysis method (27). Additional sensitivity analyses included MR-Egger, weighted median, maximum likelihood (ML), constrained ML and model averaging (cML-MA), MR pleiotropy residual sum and outlier (MR-PRESSO), and MR Steiger test of directionality (28–31). Pleiotropy was detected via the MR-Egger intercept test, where *P* > 0.05 indicated the absence of horizontal pleiotropy. The MR-Egger approach yields estimates after accounting for horizontal pleiotropy; however the statistical power is weak (28). When valid instrument variants account for more than 50% of the weight, the weighted median technique can produce consistent estimate (29). The ML method assumes that there is no pleiotropy or heterogeneity; if this assumption holds, the estimated results are unbiased (30). The cML-MA approach can be used to control for related and unrelated pleiotropy (26). The MR-PRESSO method can identify outliers and potential pleiotropy, and provide the same results as an IVW after removing outliers (31). The MR Steiger test of directionality was used to test whether the hypothesis that exposure caused the outcome was true (26). Heterogeneity among the different instrumental variables was evaluated using Cochran’s Q test. Burgess’s online calculator was used to calculate the power of the MR estimates (32).

Previous studies have found that a genetic predisposition to systolic blood pressure (SBP), diastolic blood pressure (DBP) or type 2 diabetes mellitus (T2DM) increases the risks for HDP, PE, and GH (33, 34). Therefore, we performed a multivariate MR (MVMR) analysis to estimate the direct effects of HDP, PE, and GH on stroke, especially for any significant exposure-outcome correlation identified in the univariate MR analysis (*P_IVW_* <0.05) (35). Our primary analysis used the IVW technique, and the sensitivity analyses for MVMR used MR-Egger, MR-LASSO, and MVMR-robust. MR-lasso and MVMR-robust methods can provide valid estimates with minimal bias and reduce the type I error risk in the presence of pleiotropic SNPs (36, 37). Cochran’s Q test and the MR-Egger test were used to detect heterogeneity and horizontal pleiotropy, as described previously. The strength of the instrumental variables was evaluated using the Sanderson-Windmeijer conditional F-statistic. Weak instrumental variables were identified in the MVMR analysis if the F-statistic was less than 10 (12).

The Bonferroni method was used to adjust the significance threshold. For the univariate MR analysis, the significance threshold was set to 2.38×10^-3^ (calculated as 0.05/21, with 3 exposures and 7 outcomes, totaling 21 analyses). P-values between the adjusted threshold for significance and 0.05 were considered suggestive of an association between exposure and outcome. For the MR analysis, the estimates from different MR methods were in the same direction and considered significant, at least when the IVW method estimates were significant. For the MVMR analysis, the significance threshold was set at 0.05. The association results are presented as odds ratios (OR) with 95% confidence intervals (CI).

### LD score regression analysis

Genetic correlations between HDP and stroke phenotypes were estimated by LD score regression (LDSC) (12). This analysis leveraged genetic covariance and the LD score to determine the genetic correlation between 2 phenotypes based on GWAS summary-level data. Here, we used the ‘ldscr’ R package to assess genome-wide pairwise associations between 2 different traits (38). A strong correlation was considered when the genetic correlation coefficient (*rg*) was greater than 0.7 The significance threshold for the LDSC analysis was set to 2.38×10^-3^ (0.05/21).

R software (version 4.2.2; R Foundation for Statistical Computing, Vienna, Austria) was used for all statistical analyses. The R package for MR analysis consists of the TwoSampleMR (version 0.5.6), MVMR (version 0.3), MR-PRESSO (version 1.0), and ldscr (version 0.1.0) (31, 38, 39).

## Results

### Univariate MR

**Figure 2:**
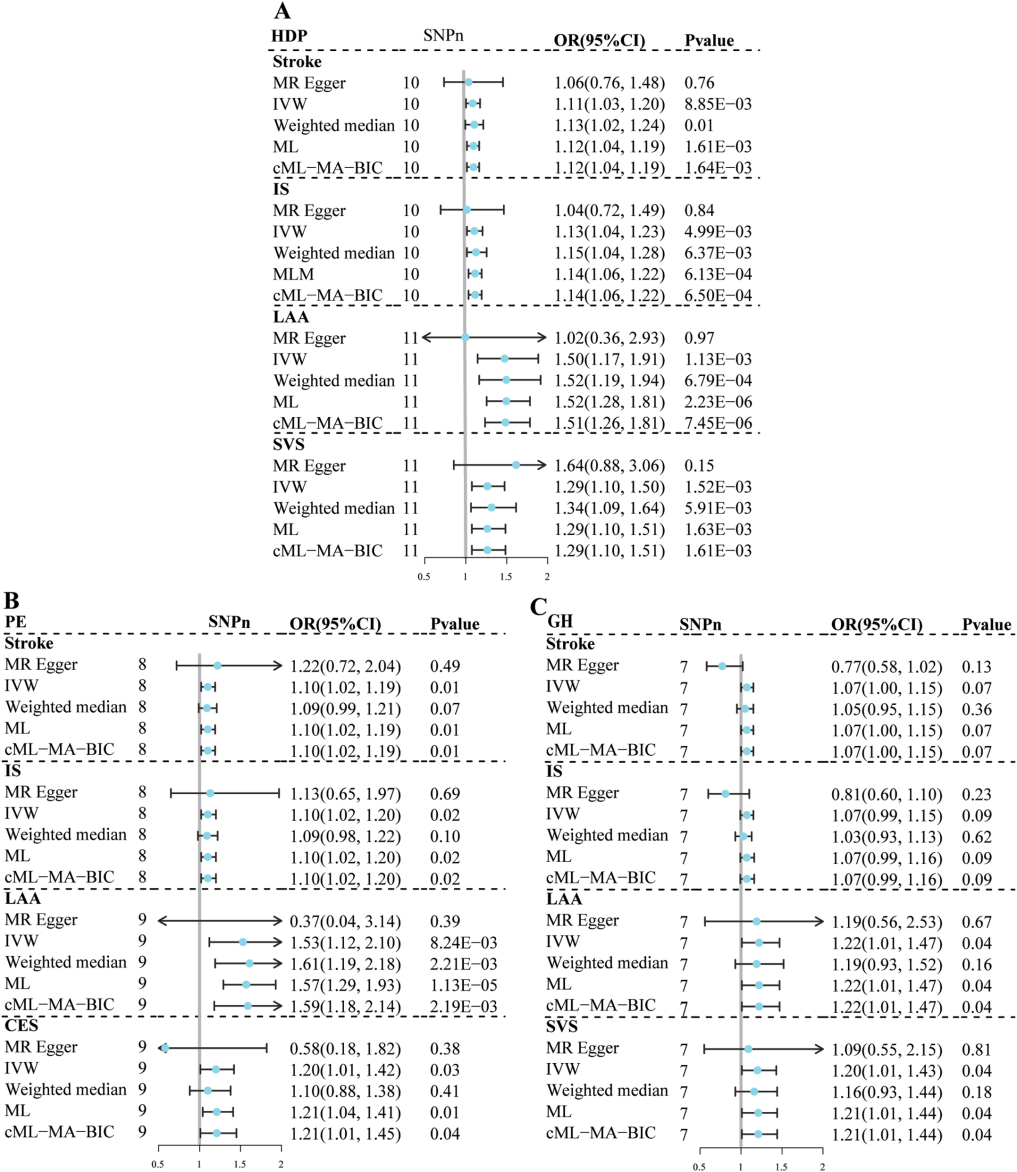
Univariate Mendelian randomization analysis for the effect of hypertensive disorders of pregnancy and stroke subtypes. **(A)** HDP; **(B)** PE; **(C)** GH. MR, mendelian randomization; HDP, hypertensive disorders of pregnancy; PE, preeclampsia or eclampsia; GH, gestational hypertension; IS, ischemic stroke; LAA, large artery atherosclerosis; SVS, small vessel stroke, CES, cardioembolic stroke; IVW, inverse variance-weighted multiplication; ML, maximum likelihood; CML-MA-BIC, constrained ML and model averaging and Bayesian amount of information; MR-PRESSO, MR pleiotropy residual sum and outlier; SNPn, number of single-nucleotide polymorphism; OR, odds ratio; CI, confidence interval.

Thirteen, 9, and 9 SNPs were selected as instrumental variables for HDP, GH, and PE, respectively (**Supplementary Table S2**). The F-statistic for each instrument was greater than 10, indicating sufficient instrumental strength. The details of the SNPs associated with exposure to the outcomes are provided in **Supplementary Table S3.** Univariate MR results for the effects of HDP, PE, and GH on stroke are shown in **Figure 2**; **Supplementary Table S4**.

Genetically predicted susceptibility to HDP was associated with an increased risk of LAA (OR=1.50, 95% CI: 1.17–1.91, *P*=1.13×10^-3^) and SVS (OR=1.29, 95% CI: 1.20–1.50, *P*=1.52×10^-3^); and possibly IS (OR=1.13, 95% CI: 1.04–1.23, *P*=4.99×10^-3^) and stroke (OR=1.11, 95% CI: 1.03–1.20, *P*=8.85×10^-3^) based on the IVW method. The remaining 4 sensitivity analyses yielded consistent estimates in the same direction. No evidence of heterogeneity, horizontal pleiotropy, or outliers was found in the remaining analyses, except for the LAA analysis had heterogeneity. No associations were found between HDP and CES, lacunar infarction and ICH.

Genetically predicted susceptibility to GH may be associated with an increased risk of LAA (OR=1.22, 95% CI: 1.01–1.47, *P*=4.07×10^-2^) and SVS (OR=1.20, 95% CI: 1.01–1.43, *P*=3.74×10^-2^). No evidence of heterogeneity or horizontal pleiotropy was observed. Although the MRPRESSO test suggested potential horizontal pleiotropy (MRPRESSO Global Test: *P*=1.00×10^-2^), no outliers were detected in the LAA analysis. In addition, the results of the ML-based and cML-MA methods were consistent with those of the IVW method. No association was found between the genetically predicted GH susceptibility and stroke or other stroke subtypes.

Genetically predicted susceptibility to PE may be associated with an increased risk of stroke (OR=1.10, 95% CI: 1.02–1.19, *P*=1.16×10^-2^) and IS (OR=1.10, 95% CI: 1.02–1.20, *P*= 1.84×10^-2^). The remaining 4 sensitivity analyses yielded consistent estimates in the same direction. No evidence of heterogeneity, horizontal pleiotropy, or outliers were observed. In the association analysis between PE and the LAA, SVS, or CES, there were suggestive associations based on the IVW method. Although the results of the ML and cML-MA methods were consistent with those of the IVW method, the variations in the conclusions obtained using the MR-Egger method and the heterogeneity and potential horizontal pleiotropy indicated the estimates were not robust.

In all univariate MR analyses, the Steiger test results suggested that the direction of causality was consistent with the hypothesized direction (**Supplementary Table S5**). The statistical power was ≥80% in most of the MR analyses regarding associations between HDP and stroke.

### Multivariable MR

**Figure 3:**
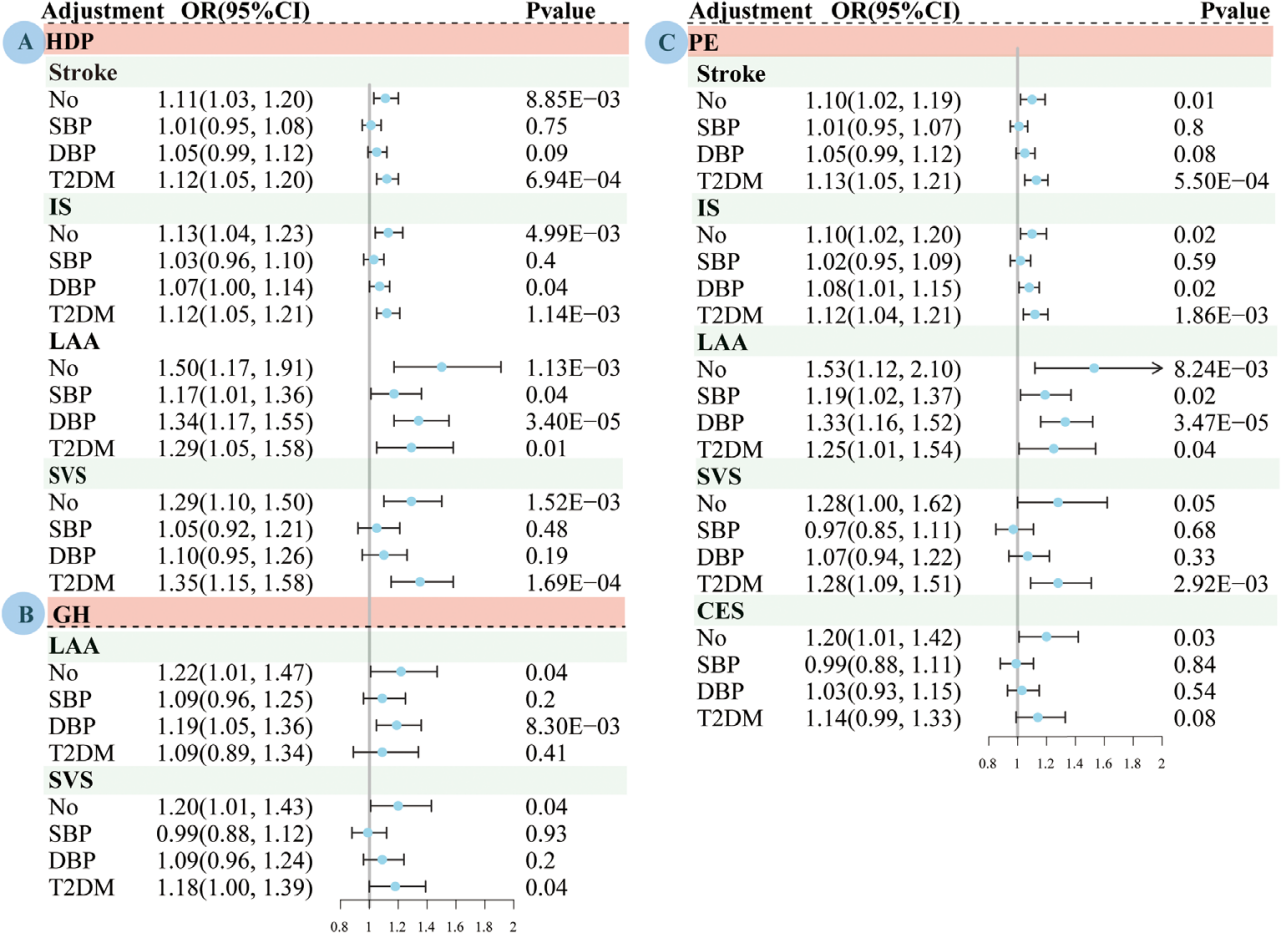
Multivariate Mendelian randomized analysis of the effects of adjustment for systolic blood pressure, diastolic blood pressure and type 2 diabetes on pregnancy induced hypertension and stroke subtypes based on the IVW method. **(A)** HDP. **(B)** GH; **(C)** PE. MR, mendelian randomization; HDP, hypertensive disorders of pregnancy; PE, preeclampsia or eclampsia; GH, gestational hypertension; IS, ischemic stroke; LAA, large artery atherosclerosis; SVS, small vessel stroke, CES, cardioembolic stroke; IVW, inverse variance-weighted multiplication; SBP, systolic blood pressure; DBP, diastolic blood pressure; T2DM, type 2 diabetes mellitus; OR, odds ratio; CI, confidence interval.

The MVMR results of the effects of HDP, PE, and GH on stroke are presented in **Figure 3**; **Supplementary Tables S6**-**S8**. After adjustment for SBP, the results of MVMR suggested that genetically predicted susceptibility to HDP (OR=1.17, 95% CI: 1.01–1.36, *P*=3.56×10^-2^) and PE (OR=1.19, 95% CI: 1.02–1.37, *P*=2.26×10^-2^) was associated with an increased LAA risk.

After adjustment for DBP, the MVMR results suggested that genetically predicted susceptibility to HDP (OR=1.07, 95% CI: 1.00–1.14, *P*=3.08×10^-2^) and PE (OR=1.08, 95% CI: 1.01–1.15, *P*=1.51×10^-2^) were associated with an increased risk of IS; and HDP (OR=1.34, 95% CI: 1.17–1.55, P=3.40×10^-5^), PE (OR=1.33, 95% CI: 1.16–1.52, P=3.47×10^-5^) and GH (OR=1.19, 95% CI: 1.05–1.36, P=8.30×10^-3^) were associated with an increased LAA risk.

After adjusting for T2DM, the MVMR results suggested that genetic susceptibility to HDP or PE was associated with an increased risk of stroke, IS, LAA, or SVS, and that GH was associated with an increased SVS risk.

The instrumental variable strength was significantly reduced in the MVMR compared to that in the univariate MR analysis. There was evidence of heterogeneity in almost all relationships. After adjusting for SBP and DBP, the evidence of pleiotropy was tested in the association analysis between PE and LAA. Therefore, we used the MR-lasso and robust MVMR methods to correct for the bias caused by weak instrumental variables or pleiotropic SNPS. The sensitivity analyses had similar estimation results for most MVMR analyses.

### LDSC analysis

**Figure 4:**
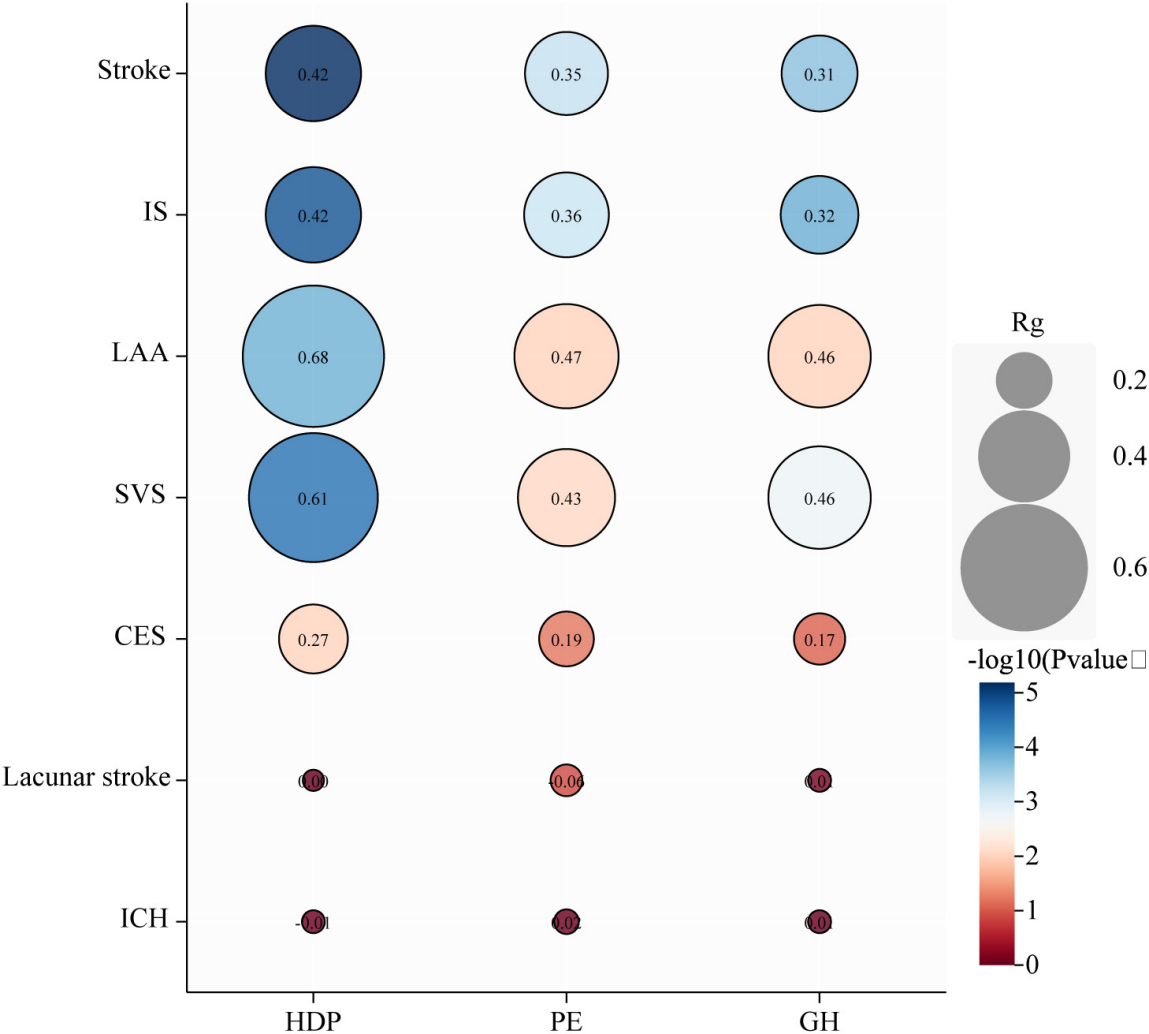
Genetic correlation analysis between hypertensive disorders of pregnancy and subtypes. HDP, hypertensive disorders of pregnancy; PE, preeclampsia or eclampsia; GH, gestational hypertension; IS, ischemic stroke; LAA, large artery atherosclerosis; SVS, small vessel stroke, CES, cardioembolic stroke; ICH, intracranial hemorrhage; *rg*, genetic correlation.

We found genetic associations between HDP, PE, and GH and stroke, IS, LAA, and SVS (**Figure 4**; **Supplementary Table S9**). No genetic associations were observed between HDP, PE, or GH and CES, lacunar infarction, or ICH.

## Discussion

We conducted a 2-sample MR study using data from major consortia and genetic studies to investigate the association between HDP and stroke risk. In the univariate analysis, genetically determined HDP, GH, and PE were associated with most stroke outcomes. In the multivariate models adjusted for SBP, the remaining tested associations were attenuated to null, except for HDP and PE, which were associated with LAA. In the multivariate models adjusted for DBP, associations, except IS and LAA outcomes, were attenuated to 0. Most associations remained significant in the multivariate models adjusted for T2DM. Our study provides genetic evidence that HDP as a risk factor for stroke.

In recent years, there has been an increasing need to evaluate HDP effects on maternal stroke (40). Several studies found that compared to normotensive pregnant women, pregnant women with HDP have an increased risk of stroke later and even in the long term (11, 41–45). A recent large, long-term study also found that women with a history of HDP had an earlier age at first stroke and an increased risk of recurrent stroke (46). Overall, our findings support the causal role of HDP in stroke pathogenesis, which is consistent with the conclusions of most observational studies. However, there is heterogeneity among the stroke subtypes. Current observational studies have mainly focused on the association between HDP or HDP subtypes, ischemic stroke and hemorrhagic stroke (41–43, 46). Few studies have explored an association among HDP, its subtypes, and ischemic stroke subtypes. Our study adds to the existing literature Mendelian randomization. MR studies have the advantage of limiting the effects of confounding factors and bias, as well as potential causal relationships (35). The results of the present study suggest that HDP as a whole is more likely to be associated with LAA and SVS, which was supported by suggestive associations between GH and PE and LAS and SVS, which were lacking in previous observational studies. Previous studies highlighted PE as an important risk factor of stroke; although HDP is a non-negligible risk factor of maternal ICH during or after pregnancy (9, 11, 47). However, in this study, only a suggestive association was found between PE and stroke and no association was found between HDP and ICH. There are several possible explanations for this null result; biases due to confounding factors, such as hypertension or other unmeasured confounders, cannot be completely excluded in observational studies; and no genetic association was observed between HDP or its subtypes and ICH, thus perhaps they do not have a causal relationship, but rather appear to be risk factors. Future observational studies are warranted to clarify the association between HDP and stroke subtypes as well as large-scale MR studies to further investigate the causal relationship between PE, GH, and stroke.

Once the correlation between HDP and stroke is understood, further exploration of the underlying biological mechanisms is important to guide and develop feasible preventive protocols to minimize stroke risk. Among the associations reported in our study, HDP was more strongly associated with stroke, whereas the strength of the association was attenuated for GH and PE. A possible reason for this is a broader HDP definition including chronic hypertension during pregnancy, was used. There may also be other underlying biological mechanisms. However, evidence emerged indicating a genetic association among hypertension, DM, and HDP. Therefore, we performed a multivariate MR analysis adjusted for hypertension and DM to determine the direct effect of HDP on stroke. After adjusting for SBP, the association between genetically determined HDP and most stroke outcomes was significantly attenuated, whereas after adjusting for diastolic blood pressure or T2DM, there was still a significant correlation. This suggests that systolic blood pressure plays a non-negligible role in the association between HDP and stroke. These results may be of interest. For example, this supports clear and feasible targets for primary prevention in multipara with a history of HDP; timely and effective control or treatment of these targets can be a key strategy for reducing stroke risk (48). Critical underlying biological mechanisms warrant further investigation. Various possible mechanisms identified in previous studies support this idea, including blood-brain barrier leakage, cerebrovascular endothelial dysfunction, neurogenic inflammatory response, and abnormal cerebrovascular autoregulation (49, 50).

A recent systematic review reported that higher blood pressure is consistently associated with adverse pregnancy outcomes, particularly when the blood pressure is above 140/90 mm Hg (51). They suggested that from 20 weeks of gestation, these blood pressure thresholds can help identify women who experience adverse pregnancy outcomes. Blood pressure monitoring is a simple and feasible measure for preventing stroke events in women whose blood pressure exceeds the threshold during pregnancy. The American Heart Association guidelines emphasize that women with HDP during pregnancy should be screened for cardiovascular risk factors in time (52). Recent studies suggest that patients with HDP have a significantly increased risk of being diagnosed with chronic hypertension in the first postpartum year and predict that patients with HDP will have a significantly increased incidence rate of cardiovascular diseases during the next 30 years (53, 54). Future studies should emphasize the importance of continuous follow-up of multiparas with a history of HDP (52). In conclusion, any feasible measure are aimed at reducing the risk of stroke in multiparous women with a history of HDP.

To our knowledge, this was the first MR study to discuss the association between HDP and its two subtypes, and the risk of stroke and several subtypes. Our results provide a genetic basis supporting HDP as a sex-specific risk factor for cerebrovascular disease (55). More attention should be given to the roles of hypertension and DM in the relationship between HDP and stroke. The underlying candidate mechanisms require further clarification to guide clinical management and drug development.

This MR study has several limitations. First, sex-specific GWAS data were used for exposure; however, the outcome data were sourced from pooled data for both males and females, although the initial analysis of the original outcome was adjusted for sex. Second, exposure was assessed as a binary variable in our MR study, making it impossible to explore linear relationships. Third, the GWAS summary data for PE used in this study included a small proportion of Central Asian ancestry, while the remaining summary data only included European populations, thus subject to demographic biases and limit the generalization of the findings to populations with other ancestries. Fourth, we used the most recent ICH data, which may have resulted in overlapping samples between exposure and outcomes; however, the 2-sample MR approach has been shown to be applicable in this context (56). Fifth, although we used multiple sensitivity analysis methods to obtain the least biased estimates, the results should be interpreted with caution due to potential pleiotropy or the introduction of weak instrumental variables in the MVMR analysis. Finally, the effect of HDP on other stroke subtypes cannot be overlooked in this investigation, because a 2-sample MR may bias false negative findings (57). Future research should identify an approach to overcome the limitations of this current dataset to clarify the relationship between HDP and stroke.

## Conclusion

This MR study provides evidence that HDP was associated with a high risk of stroke, specifically for LAA and SVS. Hypertension and T2DM play important roles in stroke risk for multiparous women. Overall, these results provide supplementary evidence supporting the possibility that HDP is a sex-specific risk factor for cerebrovascular disease. The modifiable risk factors are important targets for primary prevention in women with HDP history. Preventive interventions in pregnant patients with HDP may be required to reduce their long-term stroke risk.

## Data Availability

The original contributions presented in the study are included in the article/**Supplementary Material**. Further inquiries can be directed to the corresponding author.
